# Notes and News

**Published:** 1898-07-23

**Authors:** 


### Notes and News.

(Collected and Communicated.)

A modified water famine is causing considerable distress in the east-end of Edinburgh.

A new wing, containing accommodation for the nurses and a new operating theatre, is in course of being added to the Boston Cottage Hospital.

Mr. T. McKinnon Wood distributed the prizes to the students of the Charing Cross Hospital Medical School on Wednesday last.

The official balance sheet shows that the sum of £45,000 was expended in connection with the late Maidstone epidemic. The doctors' fees amounted in all to £5,200.

The military branch of the Army and Navy Invalid and Training Homes at Portsmouth was recently opened by Lady Davis, accompanied by Sir John Davis, who commands the Southern District. Subscribers of £2 are entitled to nominate a patient, and suitable cases are admitted for periods of four weeks. Major C. de Winton is the hon. secretary, and will give any particulars asked for.

Mr. Douro Hoare, Chairman of the London Hospital Medical College, on Monday last distributed the following prizes: Price Scholarship in Science—£120, Mr. J. Jones. Price Scholarship in Anatomy and Physiology—£60, Mr. R. Warren. Entrance Science Scholarships—£60 scholarship, Mr. R. V. Dolbey; £35 scholarship, Mr. T. Williams. Buxton Scholarships (Arts)—£30 scholarship, Mr. M. Culpin; £20 scholarship, Mr. J. Rodolph. Clinical Medicine—£20 scholarship, Mr. A. F. Tredgold; honorary certificates, Mr. W. E. Heilbom and Mr. C. White; Clinical Surgery—£20 scholarship (*æq.* scholarship division), Mr. R. Howard and Mr. G. R. Slade; honorary certificates, Mr. E. Frazer and Mr. C. Pike. Clinical Obstetrics—£20 scholarship, Mr. J. Sherren; honorary certificates—Mr. R. C. B. Wall and Mr. D. J. Thomas. Andrew Clark Prize—honorary certificates, Mr. R. C. B. Wall, Mr. A. F. Tredgold, and Mr. C. White.

The usual summer-autumn rise in the prevalence of infectious diseases has commenced, the number of notifications received by the Metropolitan Asylums Board during the past fortnight having increased by 135.

The final meeting of the London Hospital Bazaar Committee was held at the London Hospital on Monday, when a cheque for £12,000 was handed to the chairman of the hospital as the proceeds of the bazaar.

Sir William Fraser has left a handsome legacy to the Edinburgh Infirmary, and Mr. A. Teacher, of Glasgow, has bequeathed £30,000 in trust in equal shares to the Glasgow Royal Infirmary, Victoria Infirmary, and the Weston Infirmary. In addition, he has left £50,000 for distribution among those infirmaries of Glasgow and other philanthropic institutions which are purely unsectarian.

At the sixth annual meeting of the Church Sanitary Association, held at the Westminster Palace Hotel, all the speakers dwelt on the necessity of the Church caring "as well for the body as the soul." The Chairman (the Rev. A. Robins) said the object of the society was "to humanise humanity, to deliver 'rookeries' from darkness and dirt, and to wage war against overcrowding, and rescue people from the shameless life of savage surroundings."

Dr. Arthur Whitelegge, H.M. Chief Inspector of Factories, has issued a notice drawing the attention of medical practitioners to the obligation laid upon them by Section 29 of the Factory and Workshops Act, 1895. Under this section every medical practitioner attending on, or called in to visit, a patient whom he believes to be suffering from lead poisoning, phosphorus poisoning, arsenic poisoning, or anthrax, contracted in any factory or workshop, is required to notify the case forthwith to the Home Office.

The Commander-in-Chief, Lord Wolseley, has expressed the fullest sympathy with the agitation set on foot by the *Morning Post* for the provision of convalescent homes for soldiers. While warmly approving the scheme, he thinks that soldiers' convalescent homes should be provided by the public rather than by the Government. He has adopted the suggestion made by the writer of the *Post* articles that part of the proceeds of the Military Tournament should be devoted to army convalescent purposes, and he has offered to support by all the means in his power any scheme which is put on foot to afford convalescent privileges to sick soldiers.

It appears that the struggle of the hospitals for funds to carry on their work is as great in Matabele as in London. The Buluwayo Hospital, despite the marvellous strides made in the material welfare of the town, received only £300 last year from public subscription. A great many of the patients are, of course, paying cases, and the troopers and miners attached to the Chartered Company are paid for by the corporation. Nevertheless, there are heavy expenses connected with the commissariat of the institution. Wages are enormous, and many free cases treated; and the hospital finance is apt to wax towards an unsoundness very trying to those who are responsible for the management of the institution.

